# Unravelling the origin of the giant Zn deficiency in wurtzite type ZnO nanoparticles

**DOI:** 10.1038/srep12914

**Published:** 2015-09-03

**Authors:** Adèle Renaud, Laurent Cario, Xavier Rocquelfelte, Philippe Deniard, Eric Gautron, Eric Faulques, Tilak Das, François Cheviré, Franck Tessier, Stéphane Jobic

**Affiliations:** 1 Institut des Matériaux Jean Rouxel, Université de Nantes, CNRS, 2 rue de la Houssinière, 44322 Nantes cedex 3, France; 2 Institut des Sciences Chimiques de Rennes, Université de Rennes 1, CNRS, 263 Avenue du General Leclerc, 35042 Rennes cedex, France

## Abstract

Owing to its high technological importance for optoelectronics, zinc oxide received much attention. In particular, the role of defects on its physical properties has been extensively studied as well as their thermodynamical stability. In particular, a large concentration of Zn vacancies in ZnO bulk materials is so far considered highly unstable. Here we report that the thermal decomposition of zinc peroxide produces wurtzite-type ZnO nanoparticles with an extraordinary large amount of zinc vacancies (>15%). These Zn vacancies segregate at the surface of the nanoparticles, as confirmed by ab initio calculations, to form a pseudo core-shell structure made of a dense ZnO sphere coated by a Zn free oxo-hydroxide mono layer. In others terms, oxygen terminated surfaces are privileged over zinc-terminated surfaces for passivation reasons what accounts for the Zn off-stoichiometry observed in ultra-fine powdered samples. Such Zn-deficient Zn_1-x_O nanoparticles exhibit an unprecedented photoluminescence signature suggesting that the core-shell-like edifice drastically influences the electronic structure of ZnO. This nanostructuration could be at the origin of the recent stabilisation of p-type charge carriers in nitrogen-doped ZnO nanoparticles.

Zinc oxide is a material of paramount importance exhibiting pigmental, (photo) catalytic, piezoelectric, antibacterial, or varistor properties^1^,^2^,^3^,^4^,^5^,^6^,^7^,^8^ that are already developed in many fields of industry. Still novel applications emerge in various domains but they often require the preliminary stabilization of a p-type ZnO counterpart to the natural n-type ZnO to be stimulated. In optoelectronics for instance^9^,^10^,^11^,^12^, the high optical transparency of ZnO thin films coupled with their high electrical conductivity and their strong room temperature luminescence could indeed open up the door to revolutionary technologies as light emitting diodes, lasers, etc. Unfortunately the lack of p-type ZnO slows down the launch of this promising new market activity.

On account of its valuable technological interest, many studies have been devoted to ZnO, and especially to the formation of local and extended defects to produce p-typeness1,2^13^. In short, all experiments and first principle calculations carried out on ZnO bulk agree on the difficulties in stabilizing large amount of Zn vacancies^14^,^15^,^16^,^17^, while such defects and their complexes are expected to play a pivotal role in the generation of p-type charge carriers^13^. Moreover, extensive theoretical investigations clearly stipulate that nitrogen, that is considered so far as the most natural substituent for oxygen to trigger the appearance of p-typeness in ZnO, cannot lead to p-type conductivity at ambient conditions because of too deep acceptor levels^18^,^19^,^20^,^21^. These assertions clearly point out the recurring problem in engendering p-type ZnO in a reproducible way. In that context, our recent discovery of nitrogen-doped zinc-deficient ZnO nanoparticles that clearly exhibit p-typeness for periods longer than 2 years and half on samples stored at ambient conditions is very puzzling^22^. In addition to the nitrogen doping, the main characteristic of this ZnO:N species concerns its exceptionally high concentration of zinc vacancies, as great as 17%, i.e. a so high value that the wurtzite structure should collapse. To unravel this enigma, we have (re) investigated the synthesis of undoped zinc-deficient zinc oxide nanoparticles. Our study highlights that wurtzite-type ZnO nanoparticles containing a very large amount of Zn vacancies can successfully be synthesized by decomposition of zinc peroxide (ZnO_2_) in air at low temperature. The concentration is directly controlled by the surface/volume ratio (i.e. the nanoparticle radius R) of as-obtained materials. This experimental evidence as well as DFT calculations support the formation of nanostructured spheres with zinc vacancies concentrated at the surface. Photoluminescence spectra of these deficient ZnO nanoparticles exhibit unknown features suggesting that their peculiar surface states strongly impact on their electronic structure.

## Results and Discussion

**Figure 1:**
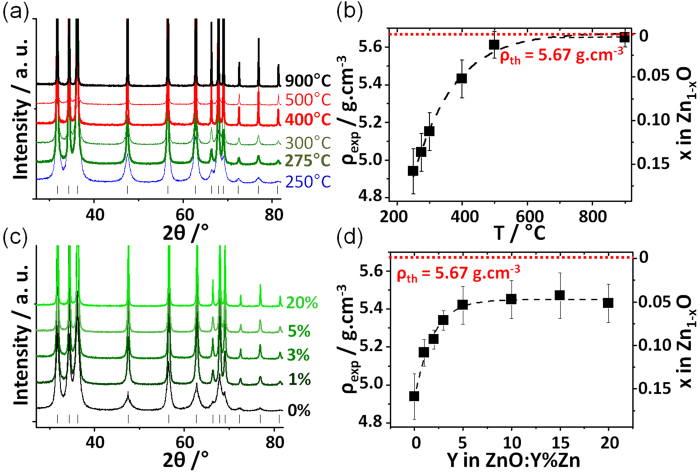
X-Ray diffraction patterns of ZnO-T_f_ (**a**) and ZnO:Y%Zn (**c**) samples (see text for definition) and evolution of the volumic mass density of the aforementioned samples with T_f_ (**b**) and Y (**d**).

Zinc peroxide, ZnO_2_, decomposes at low temperature (210°C) in air to produce white wurtzite-type ZnO nanoparticles^23^,^24^. Such a reaction occurs in an autogenous oxygen-rich atmosphere resulting from the bond breaking of peroxide groups. These conditions are expected to be favourable to the stabilization of Zn-poor ZnO. To ascertain this assertion, we synthesized ZnO samples (hereafter labelled ZnO-T_f_) by heating ZnO_2_ for 30 minutes in air at temperatures T_f_ of 250, 275, 300, 400, 500 and 900° C. Figure 1a displays the powder X-ray patterns of the as-prepared samples. No side product was detected and all the diffraction peaks were assigned unambiguously to zinc oxide with the wurtzite structure type. However, it is worth noting that Rietveld refinements (see Figure S1) carried out on ZnO materials prepared at low temperature (T_f _< 300 °C) converged systematically towards a site occupancy factor (s.o.f) at Zn site significantly lower than 1 while no deviation from the ZnO stoichiometry was noticed for materials prepared at high temperature. Hence, for ZnO-250, a Zn_0.83(2)_O chemical formula is witnessed, i.e. a ZnO structure with ≈17% zinc vacancies. To check the veracity of such a Zn deficiency, density measurements (ρ) were undertaken for ZnO-T_f_ samples using a pycnometer under He pressure. Evolution of ρ versus T_f_ is displayed in Fig. 1b. A continuous density raise depending on the synthesis temperature is clearly evidenced with a steep increase around 300 °C. Namely, density shifts from 4.94 g/cm^3^ at 250 °C, to 5.15 g/cm^3^ at 300 °C, 5.43 g/cm^3^ at 400 °C, 5.62 g/cm^3^ at 500 °C, and 5.65 g/cm^3^ at 900 °C, the latter value being very close to the theoretical one (5.67 g/cm^3^) for a hypothetical stoichiometric ZnO^25^. Really, in good accordance with XRD analyses, the exceptional low density for ZnO materials prepared at low temperature can be viewed as a clear signature of the presence of a huge amount of zinc vacancies. The number of zinc vacancies (x) in Zn_1-x_O calculated from the experimental density decreases rapidly with increasing T_f_ and almost vanishes at high temperature; x(T_f_) is indeed decreasing from 15.8% for ZnO-250 to 11% for ZnO-300, 4.6% for ZnO-400 and to less than 1% for ZnO-500 (see Fig. 1 and Table S1 in Supporting Information for details). All these results demonstrate therefore that the decomposition of zinc peroxide in air leads to Zn-poor ZnO samples with a large amount of Zn vacancies at low temperature (<300 °C), and to a ZnO without detectable Zn vacancies at high temperature (e.g. T > 900 °C) for which other type of defects such as oxygen vacancies may prevail^26^. This result is fully consistent with our previous investigations of the decomposition of zinc peroxide under ammonia which also evidenced a large amount of Zn vacancies when prepared below 300 °C^22^. Moreover the presence of Zn vacancies in ZnO nanoparticles prepared under low temperature conditions was recently reported by another group^27^.

**Figure 2:**
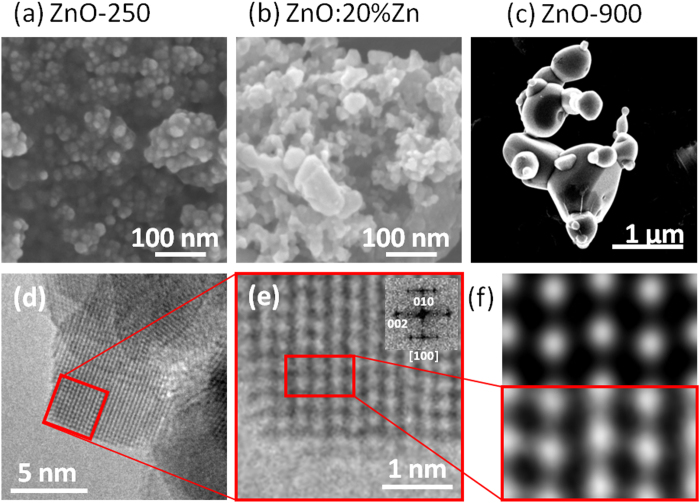
SEM images of ZnO-250 (**a**), ZnO:20%Zn (**b**) and ZnO-900 samples (**c**). TEM micrographs of ZnO-250 (**d**), (**e**). Inset in (**e**) is the corresponding FFT. Spots and [100] zone axis were identified with the stoechiometric ZnO structure (ICSD file: 162843). (**f**) Top: simulated image (thickness 3.25 nm, defocus 30 nm), bottom: experimental micrograph.

**Figure 3:**
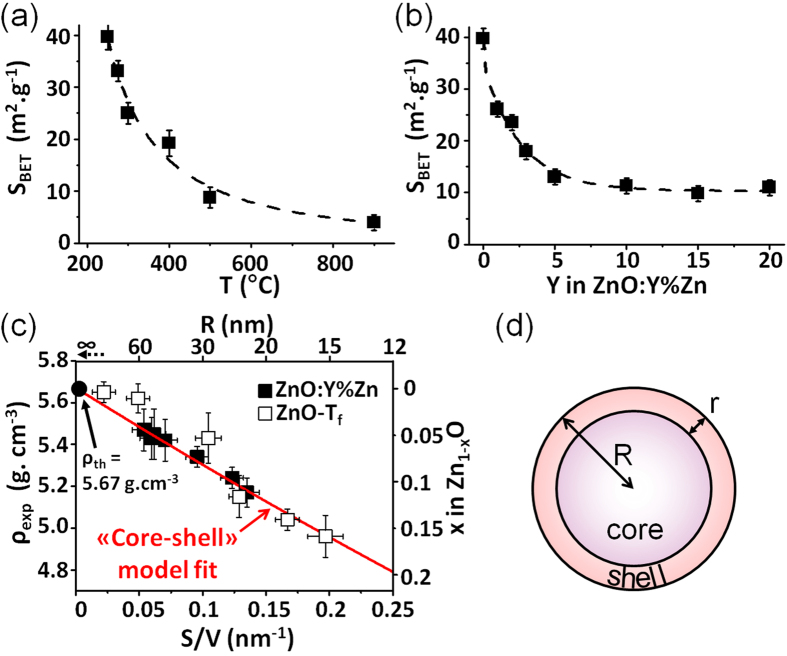
Evolution of the specific surface area (S_BET_) of the ZnO-T_f_ and ZnO-Y%Zn samples with the synthesis temperature (**a**) and the amount of zinc nitrate (**b**), respectively. Evolution of experimental densities of all prepared ZnO samples versus their Surface/Volume ratio (**c**). Scheme of the spherical core shell like model proposed for the Zn deficient ZnO particles with a radius R and a shell r (**d**). The red line in (**c**) represents the best fit of the density versus S/V data obtained using this core shell model.

At this stage, it appeared natural to wonder whether the Zn vacancies stabilized in Zn-poor samples prepared at low temperature (e.g., ZnO-250) could be filled continuously in a controlled way with divalent cations. In that framework, we have performed additional syntheses starting from mixtures of zinc peroxide and zinc nitrate. These mixtures, namely (1-Y) ZnO_2_ – Y Zn(NO_3_)_2_,6H_2_O (samples hereafter labelled ZnO:Y%Zn), were heated at 250 °C for 30 minutes. As observed previously, powder X-ray analyses revealed the presence of wurtzite-type zinc oxide without impurity (see Fig. 1c). In parallel, density measurements for Y values ranging from 0 to 20 at% pointed out the drastic impact of zinc nitrate on the chemical composition of synthesized materials (see Fig. 1d and Table S2 in Supporting Information). Clearly, the addition of nitrates, even in small amounts (e.g. 1 or 5 at%), is sufficient to increase the density of the final product from 4.94 g/cm^3^ to about 5.43 g/cm^3^, thus shifting the chemical composition from Zn_0.842_O to Zn_0.947_O, respectively. Here also, these compositional changes are concomitant with an increase of the s.o.f. calculated for Zn (from 0.83 to 0.87 and 0.95 for Y = 0, 1 and 5, respectively) from powder X-ray Rietveld refinements. Beyond Y = 5%, density tends asymptotically towards 5.45 g/cm^3^, i.e. the Zn_0.951_O composition. Thus, it may be concluded that zinc nitrate reacts with zinc peroxide to eradicate 70% of the zinc vacancies present in ZnO-250 prepared in the same conditions. The theoretical density of 5.67 g/cm^3^ for a 1:1 stoichiometry is not reached because temperature remains fixed at 250 °C, apparently a too low temperature to trigger the formation of the conventional ZnO. Consequently, based on these experiments, we may conclude that the addition of Zn(NO_3_)_2_,6H_2_O to ZnO_2_, as temperature, is a potential lever to fine-tune the non stoichiometry in Zn_1-x_O in a limited range depending on the synthesis temperature. A striking similarity between the effect of temperature and the addition of nitrates appears when comparing the X-ray powder patterns displayed in Fig. 1a,c. Indeed, an increase of the temperature as well as an increase of the amount of nitrates reduces the width at half maximum of the diffraction peaks which could go along with an enhancement of the crystallite size. In that context, TEM and SEM images were collected on the different samples to gain insight on the morphological aspect of the particles. Micrographs of ZnO-250, ZnO-900 and ZnO:20%Zn are displayed in Fig. 2. ZnO-250 consists of well crystallized nanospheres with diameter of 5–30 nm inherited from the precursor (Fig. 2a). At the opposite ZnO-900 exhibits well shaped micron size particles (see Fig. 2c), which illustrates that the average particles size increases with temperature. On the other hand, the ZnO:20%Zn sample prepared at 250 °C is composed of particles with widely dispersed diameters (namely from 10 to 100 nm, see Fig. 2b) that are much larger or much smaller than the average particle size found in ZnO-250 and ZnO-900, respectively. This illustrates that the average particles size increases with the zinc nitrate concentration Y for ZnO:Y%Zn samples. To confirm these trends, specific surface areas (S_BET_) were then measured using the Brunauer-Emett-Teller method. Figure 3a,b display both the variations of S_BET_ versus T_f_ and Y, respectively. In both cases, an exponential like decrease was observed, namely from 40 m^2^/g to 4 m^2^/g and 11 m^2^/g for ZnO-250 to ZnO-900 and ZnO-20%, respectively (see Tables S1 and S2 in Supporting Information for details). These experiments undoubtedly attest that nitrate addition plays a similar role to that of temperature and trigger the growth of particles which may explain why S_BET_ and density evolve in the same way with nitrate concentration or T_f_. Such results lead us naturally to conclude that the main parameter driving the density of zinc oxide nanoparticles, and therefore the amount of zinc vacancies, is their size.

In order to check this assumption, the density and the off-stoichiometry x parameter in Zn_1-x_O are plotted in Fig. 3c as a function of the S/V ratio, calculated using the relationship S/V = S_BET_ x ρ. Note that in case of spherical particles this S/V ratio is directly proportional to the reciprocal radius (1/R) of the spheres. A quasi linear relationship is observed between the density and S/V ratio. This confirms that zinc deficiency in zinc oxide prepared from the decomposition of ZnO_2_ is directly related to the mean size of the nanoparticle and the S/V ratio. The smaller the particle, the larger is the amount of zinc vacancies. This clearly suggests that zinc vacancies are preferentially located in the vicinity of the surface of the nanospheres what would lead to a core-shell-like model built upon a regular dense ZnO core coated by a Zn-poor zinc oxide shell. Figure 2d,e display high resolution TEM micrographs of ZnO-250 along the [100] axis. They reveal that the nanoparticles are perfectly crystallized with very regular atomic stacking up to the surface. These images do not evidence any kind of disorder and defects. Moreover, the atomic layer contrasts observed on this image can be reconstructed taking the stoichiometric ZnO-wurtzite structure as structural model (Fig. 2f). No indication of a shell of a few atomic layers thick is detected which suggests that the zinc vacancies would be located at the surface.

**Figure 4:**
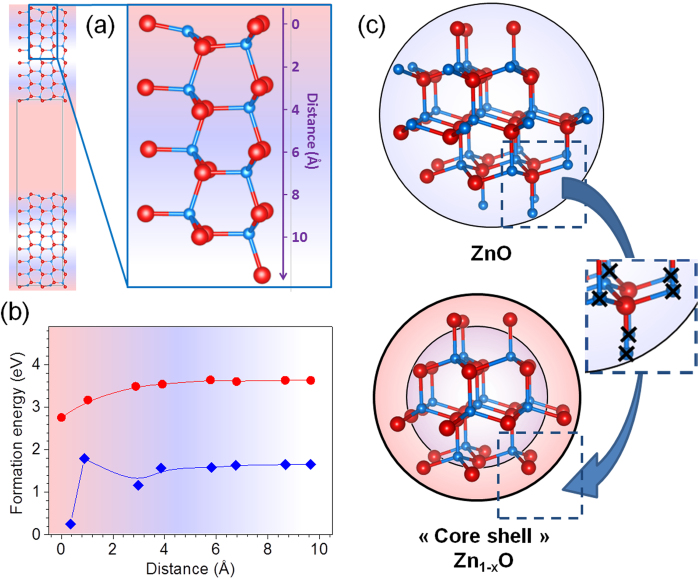
Schematic representation of the ZnO slab used to estimate from DFT calculations the energy formation of Zn and O vacancies. (**a**). The distance of a Zn atom from the surface of the slab is represented in the zoom. Evolution of the formation energy of Zn (blue circles) and O (red circles) vacancies versus their distance from the surface of the ZnO slab in oxygen-rich conditions (**b**). Schematic representation of the core-shell-like model of ZnO nanoparticles illustrating the formation of Zn vacancies at the surface of the nanoparticle (**c**).

The concentration of zinc vacancies close to the surface is somehow not so surprising. First theoretical calculations have already shown that creating large amounts of zinc vacancies in bulk ZnO is very unfavorable to an energetic point of view^14^,^16^,^17^. Second, we may expect that under overall oxidizing synthetic conditions, oxygen-terminated surfaces are privileged over zinc-terminated surfaces for passivation reasons. In order to confirm this assumption, we have performed first-principles calculations on the non-polar ZnO (100) surface using a slab geometry of periodically repeated 8a × 3b × 2c supercells containing 8 atomic planes (Zn_96_O_96_, Zn_95_O_96_ and Zn_96_O_95_ chemical formulations for stoichiometric, Zn-poor and Zn-rich ZnO samples, respectively) (Fig. 4a). From these calculations, we have estimated the formation energies of V_Zn_ (and V_O_) for the present slab geometry (see Supplementary Information for details). As displayed in Fig. 4b, under oxygen-rich atmosphere, i.e. our experimental conditions, the formation of zinc vacancies is much more favourable than the formation of oxygen vacancies. Our GGA-PBE calculations reveal an uncorrected energy of formation of zinc vacancies of 1.66 eV which is consistent with the value of 1.46 eV reported by Kohan *et al.* using LDA^28^ (see Supplementary Information for details). But the most striking feature disclosed by our calculations is that the creation of a zinc vacancy costs less energy if located at the surface than in the bulk. In our calculations, the removal of a 3-fold coordinated zinc atom at the surface requires 1.4 eV less than the removal of a 4-fold coordinated zinc atom in the bulk. This supports our assertion that the thermal decomposition of ZnO_2_ would trigger the formation of core-shell-like spherical nanoparticles consisting of a core made of an almost stoichiometric ZnO material and a shell made of a dense oxygen monolayer (or more likely an oxohydroxide monolayer see below). Such a scenario, schematized in Fig. 4c, provides a straightforward explanation for the formation of Zn poor ZnO nanoparticles with a density depending on the S/V ratio (i.e. the reciprocical radius of the nanospheres). Based on this simple model we can easily mimic the evolution of density versus the S/V ratio and fit the experimental data. Indeed considering the density of the core as the theoretical density of ZnO (5.67 g/cm^3^) and the density of the shell as the one of a close packing of oxygen (1.12 g/cm^3^), the density of the particles follows the equation (1):
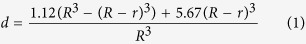
with R the radius of the spherical particles (R =3V/S), and r the thickness of the surface coating (see Fig. 3d).

**Figure 5:**
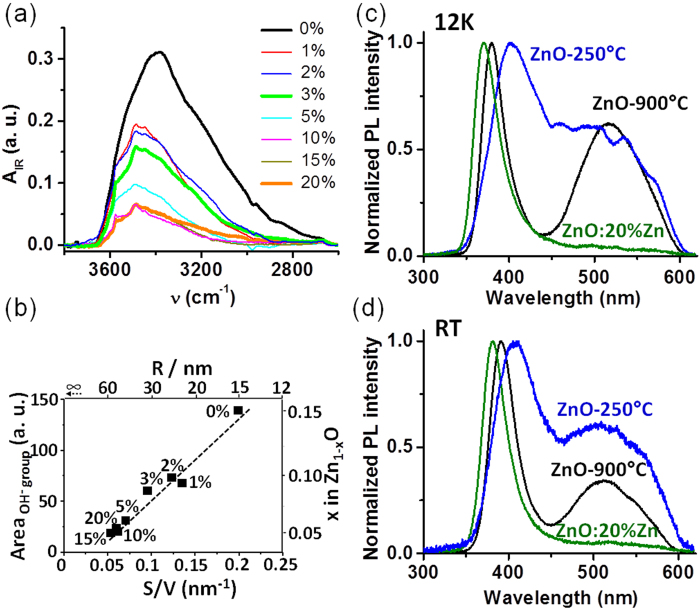
IR spectra in the 3800-2600 cm^−1^ range of the ZnO:Y%Zn samples for Y = 0, 1, 2, 3, 5, 10, 15 and 20. (**a**). Evolution of the intensity of OH bands vs. the S/V ratio (**b**). Transient photoluminescence spectra of ZnO-250, ZnO-20%Zn and ZnO-900 at 12 K (**c**) and at room temperature (**d**).

The examination of Fig. 3c highlights the very good matching between experiments and calculations for the whole series of ZnO:Y%Zn and ZnO-T_f_ materials. Moreover the fit allows estimating the value of the shell thickness as this is the only free parameter of the calculation. The r value is calculated at ≈8 Å. This value is clearly overestimated mainly because the nanoparticles are supposed to be fully isolated in our model (no contact to each others) what is far away from the reality. Namely, the mean radius of the particles calculated using the relationship R = 3V/S = 3/(S_BET_ x d) is overestimated by a factor of 2–3 if we compare to the real size of the particles revealed by TEM (e.g. for ZnO-250 the calculated R values is 15 nm while TEM images shown in Fig. 3d exhibits particles of R ~ 5–10 nm). This discrepancy originates from the natural propensity of particles to aggregate what reduces the measured S_BET_ and therefore increases the calculated R. In that respect, our calculation supports a core shell model with a core made of ZnO and coated by a shell of oxygen or hydroxyl groups. Moreover, chemically speaking, the presence of such hydroxyl groups are highly plausible at ambient conditions and will lead to the overall (Zn^2+^)_1-x_(O^2−^)_1-2x_(OH^−^)_2x_ charge balance. IR spectroscopic measurements were therefore undertaken on ZnO-250 and ZnO:Y%Zn samples (see Fig. 5a). They fully confirmed the presence of OH groups and clearly evidence a linear correlation between the integrated intensity of the IR absorption band at 3400 cm^−1^ and the measured S/V ratio (Fig. 5b)^29^,^30^. Namely, the lower the particle size the more intense the IR absorption. This fully supports the coating of our nanoparticles by a dense oxo-hydroxide monolayer.

PhotoLuminescence (PL) is a powerful tool routinely used to characterize the presence of defects which is very often used in case of ZnO as this compound exhibit a strong exciton around 380 nm 1^19^,^31^,^32^,^33^. In that framework, transient PL measurements were initiated on ZnO-250, ZnO-900 and ZnO:Y%Zn at 300 and 12 K to gain insights on the effect of the Zn vacancies on the optoelectronic properties of our nanoparticles. Figure 5 shows that the PL spectra of all compounds present a strong band around 380 nm due to the free exciton recombination, and a weaker, broader feature centered around 500 nm which is characteristic of structural defects. First, there is a shift of the free-exciton band depending on the nature of the material. In Zn-poor sample ZnO-250 the maximum of the free-exciton band is located at 410 nm, while those of ZnO-900 and ZnO:20%Zn appear at 390 nm and 380 nm, respectively at 12 K (Fig. 5c). The same trend is observed at room temperature (Fig. 5d), and it should be noted that the free exciton band is very broad for Zn poor sample ZnO-250. The defect band observed for ZnO-900 at 510 nm is similar to that reported in the literature and generally ascribed either to oxygen vacancies, interstitial Zn atoms, or oxide antisite defects 9^19^,^34^. On the contrary, in Zn poor sample ZnO-250 the defect band shows unprecedented features and appears as a broad and intense structured shoulder with at least 6 components between 450 nm and 550 nm. These superimposed features at different energies suggest a distribution of defects in the sample which is consistent with the presence of a large amount of Zn deficiency. This is further confirmed, by the almost complete disappearance of this defect band in the PL spectra of ZnO:20%Zn which contains much less zinc vacancies. Therefore, the presence of zinc vacancies at the surface of the zinc oxide nanoparticles affects drastically their optoelectronic properties. This gives hope that engineering the surface states of these nanoparticles may be a good way to tune their electronic properties. Our previous study on the decomposition of ZnO_2_ under ammonia gives a first hint that this strategy may be used to achieve p-typeness^22^.

To sum up, we have prepared in a reproducible way zinc oxide nanoparticles with a strong Zn deficiency ensuing from the decomposition of ZnO_2_ at low temperature. The Zn deficiency is related to the nanosized character of the particles and the strong propensity of oxide and hydroxyl groups to cover such particles in ambient conditions to passivate them. The occurrence of Zn deficiency up to 16% in the core of the wurtzite-type ZnO nanoparticles can be definitely rebutted in agreement with first principle calculations sounding the stability of such defects even in small amount. However, our work shows that this type of defects can be stable close to the surface and have a strong impact on the optical properties of zinc oxide nanoparticles. Recent report by Reynolds *et al.*^35^ postulates that V_Zn_-N_O_-H^+^ complexes could generate shallow acceptor levels. In that respect, surface engineering of N doped ZnO nanoparticles hold the promise to be able to tune their electronic properties and achieve p-typeness in a more reproducible way in the near future.

## Methods

### Synthesis

ZnO_2_ precursor was synthesized according to the chemical route proposed by Uekawa *et al.*^23^,^24^. Namely, 18.9 g of zinc nitrate (Zn(NO_3_)_2_,6H_2_O, Sigma) were dissolved in 150 ml of distilled water. 150 ml of a 0.93 M NaOH solution was then added dropwise to trigger the precipitation of Zn_5_(OH)_8_(NO_3_)_3_(H_2_O)_2_, a white compound. After three centrifugation/water washing sequences, the precipitate was dispersed in 5 ml:95 ml H_2_O_2_:H_2_O solution (H_2_O_2_ 30% Purex analyses, SDS). This one was then heated at 75 °C for 2 hours under magnetic stirring. ZnO_2_ (or most likely Zn(O_2_)_1-x_O_x_,yH_2_O according to J. Li *et al.*^36^) is then obtained as off-white, 5 nm diameter large spherical nanoparticles after 3 successive centrifugation/water washing steps followed by rinsing in ethanol. “ZnO” samples, i.e. materials with different Zn:O stoichiometric ratios but with the ZnO-wurtzite structure type, were then prepared either by heating “ZnO_2_” in air at T_f_ temperatures of 250, 275, 300, 400, 500 °C and 900 °C for 30 minutes (samples hereafter labelled ZnO-T_f_), or by heating a (1-Y) ZnO_2_ – Y Zn(NO_3_)_2_,6H_2_O mixture at 250 °C for 30 minutes (samples hereafter labelled ZnO:Y%Zn with Y = 0, 1, 2, 3, 5, 10, 15 and 20). Let us notice that precursors in both cases underwent a preheating at 190 °C for 30 minutes before firing at higher temperature to avoid a too high scattering of the final products in the furnace inherent to the very exothermic (and explosive) decomposition of ZnO_2_.

### X-ray powder diffraction

X-ray powder diffraction patterns were recorded at room temperature in the 10–90 2θ range on a Bruker D8 Advance Diffractometer using the Cu K-L_2,3_ radiation. All data treatments and refinements were carried out with the JANA2006 package [Petricek, V.; Dusek, M.; Palatinus, L.; JANA 2006 The crystallographic computing system, Institute of Physics, Praha, Czech Republic, 2006].

### Density measurements

Density measurements were carried out by pycnometry under He pressure using the AccuPyc 1330 system.

### Specific surface area

Specific surface areas were obtained by Brunauer−Emmett−Teller (BET) analyses done in a micrometrics ASAP 2010BET.

### Electron microscopy

Transmission electron microscopy was performed on a Hitachi H-9000NAR (accelerating voltage: 300 kV, Scherzer resolution: 0.18 nm). Prior to examination, the samples were ground in ethanol and dispersed ultrasonically. A drop of the suspension was deposited on a copper grid previously covered with a thin holey carbon film. Scanning electron microscopy micrographs were collected on a JEOL7600F apparatus on powder spread on a carbon tape pasted over a copper metal sample holder.

### IR spectroscopy

Fourier transform infrared absorption experiments were performed with a Bruker Vertex 70 instrument equipped with a Specac Golden Gate attenuated total reflectance accessory. The spectral resolution was 4 cm^−1^.

### Photoluminescence experiments

Time-resolved photoluminescence experiments were carried out with a regenerative amplified femtosecond Ti:Sapphire laser system (Spectra Physics Hurricane X), generating 100 fs pulses at 800 nm with a repetitive rate of 1 kHz and a power of 1 W. The laser line is frequency-tripled to obtain an excitation line λexc = 267 nm (4.65 eV). The pump energy pulse is controlled to ensure that the photon density in the sample does generate annihilation processes and sample degradation. The transient signals are spectrally dispersed into an Oriel MS260i imaging spectrograph (150 grooves/mm, f = 1/4) designed to minimize stray light with spectral resolution below 3 nm. The emission spectra are temporally analysed with a high dynamic range Hamamatsu C7700 streak camera with a temporal resolution of 5 ps. Temperature measurements were conducted by attaching the sample with indium as a heat transfer medium near a nominal-temperature AsGa diode in a vacuum loading, helium cooled, continuous flow shielded cryostat down to 12 K.

## Additional Information

**How to cite this article**: Renaud, A. *et al.* Unravelling the origin of the giant Zn deficiency in wurtzite type ZnO nanoparticles. *Sci. Rep.* **5**, 12914; doi: 10.1038/srep12914 (2015).

## Supplementary Material

Supplementary Information
