# Corrigendum: Unravelling the origin of the giant Zn deficiency in wurtzite type ZnO nanoparticles

**DOI:** 10.1038/srep14592

**Published:** 2015-09-30

**Authors:** Adèle Renaud, Laurent Cario, Xavier Rocquefelte, Philippe Deniard, Eric Gautron, Eric Faulques, Tilak Das, François Cheviré, Franck Tessier, Stéphane Jobic

Scientific Reports 5: Article number: 1291410.1038/srep12914; published online: 09032015; updated: 09302015

The original version of this Article contained a typographical error in the spelling of the author Xavier Rocquefelte, which was incorrectly given as Xavier Rocquelfelte. In addition, Affiliation 2 was omitted for this author. These errors have now been corrected in both the PDF and HTML versions of the Article.

