# Supplementary material for: Unravelling the origin of the giant Zn deficiency in wurtzite type ZnO nanoparticles
Source: Sci Rep. 2015 Sep 3;5:12914. doi: 10.1038/srep12914 (PMC4558607; doi:10.1038/srep12914)
Supplement: Supplementary Information [file srep12914-s1.pdf]

Supplementary Informations

Unravelling the origin of the giant Zn deficiency in wurtzite type ZnO nanoparticles

Adèle Renaud,[1] Laurent Cario,[,1] Xavier Rocquelfelte,[1] Philippe Deniard,[1] Eric Gautron,[1] Eric Faulques,[1] François Cheviré,[2] Franck Tessier,[2] and Stéphane Jobic[*,1]*

[1] Institut des Matériaux Jean Rouxel, Université de Nantes, CNRS, 44322 Nantes cedex 3, France

[2] Institut des Sciences Chimiques de Rennes, 35042 Rennes cedex, France

1. Results of Rietveld refinements

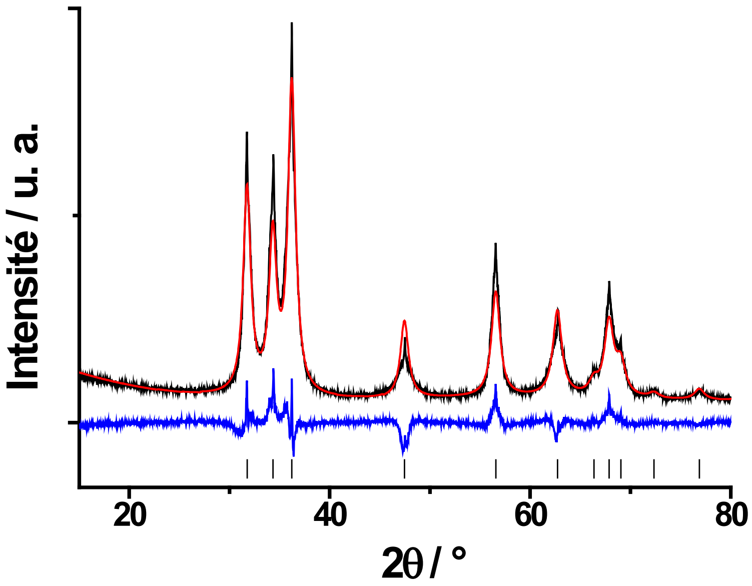

Figure S1. X-ray powder pattern Rietveld refinement performed on the powder of ZnO-250. The experimental diagram is black, the calculated diagram is red and the difference between the two is shown in blue.

2. Refined Cell volume, measured density, measured specific surface area, and calculated Zn vacancy rate for $Zn_{1-x}O$ samples.

Table S1. Refined cell volume (V), measured density (ρ), Zn vacancy rate (x) and specific surface area of $Zn_{1-x}O$ samples prepared at T_f for 30 minutes.

T_f	V (Å^3)	ρ (g. cm^{-3})	x	S_{BET}
250	47.76	4.94	0.158	39.7
275	47.69	5.04	0.138	33.1
300	47.68	5.15	0.114	25.0
400	47.66	5.43	0.053	19.2
500	47.63	5.62	0.012	8.7
900	47.65	5.65	0.004	3.9

* the x value is determined from the equation $x(Tf) = 1 - \frac{1/2(N_A.V_{Tf}.\rho_{Tf}) - M_O}{M_{Zn}}$, where N_A is the Avogadro number, V the refined cell volume of the ZnO-T_f sample (issued from a Rietveld refinement of its X-ray pattern), ρ the volumic density, and M_{Zn} and M_O the molecular weight of Zinc and Oxygen.

Table S2. Refined cell volume (V), density (ρ), Zn vacancy rate (x) and specific surface area of $Zn_{1-x}O$ samples prepared at 250°C for 30 minutes with addition of Y atomic percent of zinc nitrate.

Y	V (Å^3)	ρ (g. cm^{-3})	x	S_{BET}
0%	47.76	4.94	0.158	39.7
1%	47.68	5.17	0.110	26.1
2%	47.69	5.24	0.094	23.5
3%	47.66	5.34	0.073	17.9
5%	47.72	5.42	0.054	13.0
10%	47.66	5.45	0.049	11.3
15%	47.66	5.47	0.044	11.0
20%	47.63	5.43	0.053	9.8

* the x value is determined from the equation $x(Tf) = 1 - \frac{1/2(N_A.V_{Tf}.\rho_{Tf}) - M_O}{M_{Zn}}$, where N_A is the Avogadro number, V the refined cell volume of the ZnO-T_f sample (issued from a Rietveld refinement of its X-ray pattern), ρ the volumic density, and M_{Zn} and M_O the molecular weight of Zinc and Oxygen.

3. First principle calculations.

A 96 formula units cell has been used, i.e. 8a×3b×2c, with a, b and c the crystallographic cell parameters of ZnO. The parameters used in the VASP [1] calculations are the following. We employed the Perdew-Burke-Ernzerhof (PBE) [2] generalized gradient approximation for the exchange and correlation potential. It allows having a proper description of the structural properties of ZnO. The wave functions were expanded in a plane wave basis set with kinetic energy of 400 and 500 eV, respectively for the geometry optimization and total energy calculations. The VASP package is used with the projector augmented wave (PAW) method[3] of Blöchl. The integration in the Brillouin Zone is done by the Methfessel-Paxton method [4] on a set of k-points determined by the Monkhorst-Pack scheme [5]. All atoms were then allowed to relax by following a conjugate gradient minimization of the total energy scheme (3×10^{-2} eV/ Å).

The defect formation energies have been estimated for neutral defects using the following equation:

$$E_f = E_d - E_p - \sum_i \Delta n_i \mu_i$$

where E_d and E_p are, respectively, the total energies of the supercell containing a defect and the supercell of the perfect crystal, Δn_i is the difference in the number of constituent atoms of type I, and μ_i is the atomic chemical potential. μ_{Zn} and μ_O have been estimated using the following expression in oxygen-rich (zinc-poor) limit: $\mu_{Zn} = \mu_{Zn(bulk)} + \Delta H_f$ and $\mu_O = ½\ \mu_{O_2}$, where ΔH_f denotes the heat formation of ZnO (here we used experimental value of -3.6 eV [6]). In oxygen-poor limit (zinc-rich), these expressions are then $\mu_{Zn} = \mu_{Zn(bulk)}$ and $\mu_O = ½\ \mu_{O_2} + \Delta H_f$.

It should be noted that we have considered in this study only neutral defects. Thus it was not necessary to add correction terms to address interaction between charged defects. Our results reproduced the previous reported values for bulk-ZnO. For instance in oxygen rich-limit, we found $E_f(V_{Zn}$ bulk) = 1.66 eV using GGA-PBE, which is in good agreement with the value of 1.46 eV obtained by Kohan et al. using LDA [7]. Similarly, in oxygen-rich limit $E_f(V_{Zn}$ bulk) = 5.26 eV using GGA-PBE, which compares well with the reported LDA value of 5.47 eV. To be quantitative, additional corrections must be taken into account [8,9,10]. However these corrections remain questionable in the way they are applied and strongly impact the resulting E_f values. Here

we are interested to describe the trends between bulk- and surface-defects. We have thus neglected these additional corrections and we calculate the relative variation of formation energies of the bulk- and surface-defects in ZnO based on uncorrected DFT results.

As expected, in oxygen-rich conditions it is more favorable to form Zn vacancies, while in zinc-rich conditions it appears more favorable to form O vacancies. In both cases, the formation of a vacancy costs less energy if located at the surface. The difference between E_f(surf) and E_f(bulk) is larger for V_{Zn} than V_O. Finally, it should be noted that our samples are n-type ZnO nanoparticles, meaning that the Fermi energy is close to the conduction band bottom. In such a situation the charge state of V_{Zn} and V_O are expected to be 2- and 0 [9]. In the present calculations we found that in oxygen-rich conditions the uncorrected formation energy of V_{Zn}^{0} is about 1.66 eV in bulk-ZnO and 0.25 eV on the ZnO surface. If now we consider V_{Zn}^{2-}, an important stabilization has been reported which makes the energy of formation of this defect to be negative or slightly positive depending on the correction method [8,9,10]. This further stabilization of the V_{Zn}^{2-} compared to V_{Zn}^{0} considered in our calculations reinforces the validity of our "core shell" model. It suggests that the energy of formation of Zinc vacancies might be slightly positive in the bulk while strongly negative at the surface of the nanoparticles (see Figure S1).

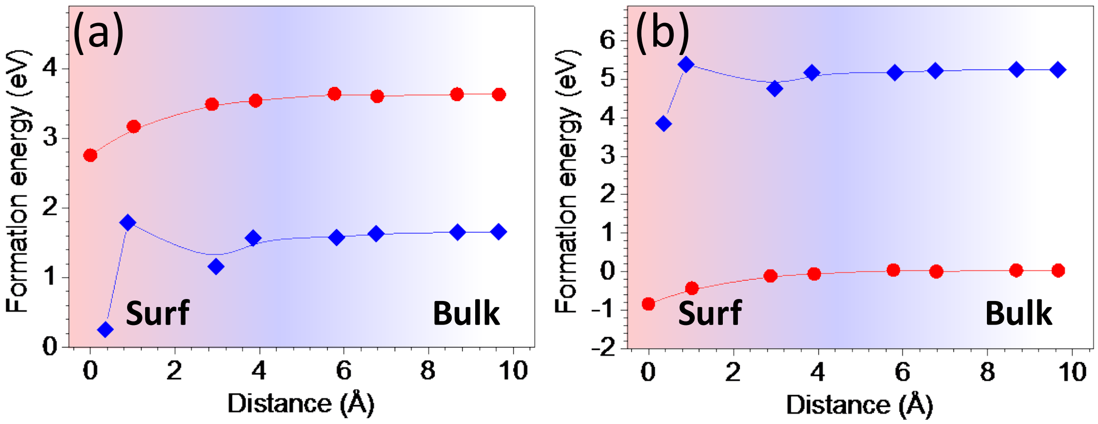

Figure S2. Evolution of the formation energy of Zn (blue circles) and O (red circles) vacancies versus their distance from the surface of the ZnO slab in (a) oxygen-rich and (b) zinc-rich conditions.

[1] Kresse, G. & Furthmuller, J. Efficient iterative schemes for ab initio total-energy calculations using a plane-wave basis set. *Phys. Rev. B* **54**, 11169-11186 (1996).

[2] Perdew, J. P., Burke, K. & Ernzerhof, M. Generalized Gradient Approximation Made Simple. *Phys. Rev. Lett.* **77**, 3865-3868 (1996).

[3] Blochl, P. E. Projector augmented-wave method. *Phys. Rev. B* **50**, 17953-17979 (1994).

[4] Methfessel, M. & Paxton, A. T. High precision sampling for Brillouin-zone integration in metals. *Phys. Rev. B* **40**, 3616-3621 (1989).

[5] Monkhorst, H. J. & Pack, J. D. Special points for Brillouin-zone integrations. *Phys. Rev. B* **13**, 5188-5192 (1976).

[6] Oba, F., Togo, A., Tanaka, I., Paier, J., Kresse, G. Defect energetics in ZnO: A hybrid Hartree-Fock density functional study, *Phys. Rev. B* **77**, 245202 (2008).

[7] Kohan, A. F., Ceder, G., Morgan, D. & Van de Walle, C. G. First-principles study of native point defects in ZnO. *Physical Review B* **61,** 15019 (2000).

[8] Oba, F., Togo, A. & Tanaka, I. Defect energetics in ZnO: A hybrid Hartree-Fock density functional study. *Physical Review B* **77,** (2008).

[9] Janotti, A. & Van de Walle, C. G. Native point defects in ZnO. *Physical Review B* **76,** 165202 (2007).

[10] Lany, S. & Zunger, A. Dopability, Intrinsic Conductivity, and Nonstoichiometry of Transparent Conducting Oxides. *Physical Review Letters* **98,** (2007).
